# Collision Detection for Underwater ROV Manipulator Systems

**DOI:** 10.3390/s18041117

**Published:** 2018-04-06

**Authors:** Satja Sivčev, Matija Rossi, Joseph Coleman, Edin Omerdić, Gerard Dooly, Daniel Toal

**Affiliations:** 1 MaREI – Marine and Renewable Energy Ireland, Cork, Ireland; matija.rossi@ul.ie (M.R.); Joseph.Coleman@ul.ie (J.C.); Edin.Omerdic@ul.ie (E.O.); Gerard.Dooly@ul.ie (G.D.); Daniel.Toal@ul.ie (D.T.); 2 Department of Electronic and Computer Engineering, University of Limerick, Limerick, Ireland

**Keywords:** underwater manipulation, collision detection, collision sensing, collision avoidance, manipulator control, robot arm, subsea inspection and intervention, marine robotics, ROV

## Abstract

Work-class ROVs equipped with robotic manipulators are extensively used for subsea intervention operations. Manipulators are teleoperated by human pilots relying on visual feedback from the worksite. Operating in a remote environment, with limited pilot perception and poor visibility, manipulator collisions which may cause significant damage are likely to happen. This paper presents a real-time collision detection algorithm for marine robotic manipulation. The proposed collision detection mechanism is developed, integrated into a commercial ROV manipulator control system, and successfully evaluated in simulations and experimental setup using a real industry standard underwater manipulator. The presented collision sensing solution has a potential to be a useful pilot assisting tool that can reduce the task load, operational time, and costs of subsea inspection, repair, and maintenance operations.

## 1. Introduction

Due to the lack of autonomous mobile-manipulator robots, tasks in remote and hostile environments are performed by manipulator arms operated by human pilots at distance. Often called telemanipulators, these devices are usually deployed to worksites onboard support base vehicles which are also remotely operated—therefore referred to as Remotely Operated Vehicles (ROVs). Work-class ROV technology has served subsea Intervention, Repair, and Maintenance (IRM) operations in various offshore industries, including oil and gas, marine construction, marine science, naval defence, and Marine Renewable Energy (MRE) [1,2,3]. Submarine work-class ROVs are generally equipped with two manipulators; one dexterous seven function manipulator that is used to perform the actual intervention task, and one simple, powerful grabber that is used to hold the ROV stationary relative to the structure on which the operation is taking place. Utilising a traditional teleoperation approach with an open-loop control system, work-class ROVs are completely reliant on the human operators who control both the ROV and the manipulator. The pilots, located on the surface vessel, acquire visual feedback of the worksite through different imaging systems and simultaneously perform various tasks by remotely controlling the manipulators’ motion with a specialised joystick [4]. ROVs are usually equipped with multiple sensing devices including camera systems, forward-looking sonars, and other sensors and tools [5]. A lot of the equipment is mounted on the front side of the ROV, therefore inside the manipulators’ workspace. ROV pilots must be extremely careful during telemanipulation not to damage the expensive equipment, the ROV’s body, the targeted structure, and the manipulators themselves. Reduced visibility due to water turbidity and poor 3D perception due to the 2D video feedback only add to the complexity of teleoperation. As a result, tasks that might seem simple can become very difficult and wearisome even for very skilled operators, significantly affecting their performance. Moreover, there is a trend towards resident ROV teleoperation of manipulators, i.e., manipulation from shore through telecommunication network infrastructure. Such a setup increases the pilot’s task load and emphasises the importance of the pilot’s skills and of the network quality, which might introduce delays in control and sensory feedback. The resident ROV teleoperation concept has recently been introduced in the industry by IKM Subsea with a permanently deployed ROV system remotely operated from shore [6]. Due to the complexities mentioned above, subsea operations are time-consuming and therefore costly. Mobilising a vessel with ROV systems onboard can cost from €18,000 per day for research vessels to well over €50,000 for oil and gas operations.

Most advanced commercial underwater ROV manipulator systems have an integrated software function to limit the range of motion of the manipulator’s joint axes. This is often done to prohibit the access to certain areas on the base vehicle and protect the equipment. However, limiting the manipulator’s motion in joint space is not efficient as it enormously limits the manipulator’s operational workspace. Moreover, it does not prevent the two manipulators from colliding, which is an important issue as these manipulators are capable of exerting considerable forces that may cause severe mechanical damage. As two manipulators with overlapping workspaces simultaneously operate in a common working area, a real-time collision avoidance algorithm is required, capable of detecting and prohibiting motion commands which would result in a collision and allowing only collision-free motion. Each manipulator represents a dynamic obstacle to the other manipulator, therefore it is necessary to address the collision between the two as well as between each of them and other obstacles.

Over the last thirty years, various researchers have been investigating collision detection methods for robotic arms, chiefly as part of collision-free motion planning algorithms [7,8,9]. Most of the research focus has been on algorithm development, evaluating them through simulations and laboratory experiments on industrial manufacturing electro-mechanical autonomous robots. Many of these approaches are off-line and designed for preprogrammed robot motion planning, and therefore not suitable for commercial ROV manipulator systems which are teleoperated utilising point-to-point control, where the full path cannot be known in advance. For this reason, any collision avoidance implementation for the teleoperated manipulators has to work on-line. There is a research trend towards automating ROV intervention operations [10], and in the case the ROV industry adopts it, off-line collision avoidance approaches might become suitable. However, this is still in its early research and development stage, and fully automated manipulator systems still do not exist in the global fleet of work-class ROVs.

Various on-line collision detection methods based on different geometrical modelling approaches have been proposed. Discretising the Cartesian space into cuboids and forming a collision map based on the obstacle-unaware trajectories has been presented by Czarnecki [11]. Greenspan and Burtnyk [12] described a method of model-based real-time collision avoidance where the manipulator links are modelled as sets of spheres and obstacles as a weighted voxel map. Henrich et al. [13] proposed an implicit and discretised configuration space (C-space)-based method where collision detection is done in the Cartesian workspace. A similar C-space obstacle boundary method based on the reachable manifold and contact manifold theories has been presented by Fei et al. [14]. Freund and Rossman [15] described a Collision Avoidance in Real-time Environments (CARE) method where the points on the robot’s surface endangered by obstacles are assigned Collision Avoidance Points (CAPs). Some authors have adopted a geometrical modelling approach where the manipulator links are modelled with spherical shells, volumes formed by moving a sphere with a certain radius on a specified primitive such as a point, line, or rectangle [16,17,18,19]. Smith et al. [20] presented a survey that includes collision detection and avoidance methods on dual-arm robots, which are kinematically identical to work-class ROVs; they are equipped with two manipulators with overlapping workspaces. Other non-geometrical model-based methods have also been investigated. Lee and Song [21] proposed a collision detection algorithm based on an external torque observer and friction model identification. This approach requires monitoring the electric current of the manipulator’s joint motors and is applicable only for electrically driven robot arms, which are rare in the ROV industry [22]. Force feedback based collision detection methods have also been proposed [23]. The issue with the last two approaches is that the collision can be detected solely after the contact has been made, and since ROV equipment includes multiple cameras with glass domes and other delicate devices, any contact is undesirable. Lumelsky and Cheung [24] experimented on whole-sensitive manipulator arms whose entire body is covered with sensitive skin capable of detecting collision with other objects. Besides the contact issue, this approach might be too complicated for underwater implementation as the sensor skin would have to be waterproof. Various machine vision methods for collision detection have been investigated [25,26,27]; however, the visibility in ROV worksites is often low, and such approaches would be condition dependent. Moreover, multiple cameras might be required to encircle the manipulators’ environment and ensure no impeding collision are missed. One of the few publications addressing collision detection for subsea ROV manipulators has been reported by Agba [28] within the “SeaMaster” ROV-manipulator system simulator, where the manipulator links are modelled using the super-ellipsoid equation and collision is tested by checking whether a point on the surface of an object lies within the inside surface of a link model.

Despite the significant achievements in academia, including many publications on autonomous subsea manipulation [29,30,31,32], collision-free manipulation has not been developed and integrated on work-class ROVs. This paper describes a real-time collision detection algorithm based on a voxel map representation developed for use on work-class ROV manipulator systems. The proposed collision avoidance mechanism is capable of sensing imminent collisions and preventing their occurrence by automatically overriding the operator’s commands and stopping the manipulator. The developed algorithm is not a collision-free path planning method capable of finding an alternative path to the one provided by the control system. In that sense, it is entirely passive, and it is left to the ROV pilot to decide on alternative routes. The developed solution is successfully integrated with the control system of a real underwater ROV manipulator. The performance of the developed approach has been validated through simulations and laboratory experiments.

The remainder of the paper is organised as follows: Section 2 presents a detailed description of the developed collision-free manipulation algorithm. Section 3 describes the simulation and experiment scenarios and presents the results. Finally, Section 4 offers some final remarks and describes future work.

## 2. Algorithm

**Figure 1:**
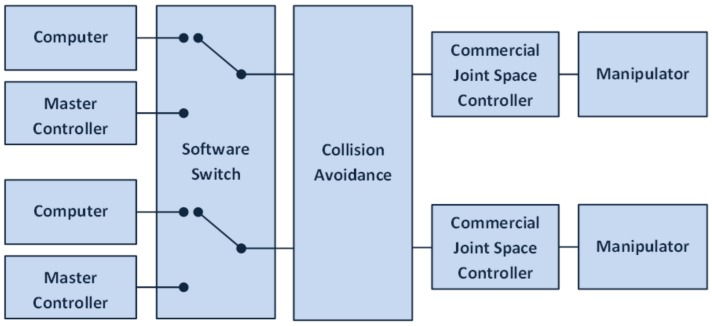
Block diagram of a dual manipulator control system.

This section describes the collision detection algorithm developed for underwater manipulators beyond the current state of the art in work-class ROV technology. The developed software is readily integrable with existing subsea hydraulic manipulator systems without any hardware or software modifications. The developed software package is running on a standard PC, located between the Master Control Unit (MCU) and the low-level joint position servo controller. A computer control software, previously developed by the authors [22], is also tested as an alternative to the traditional MCU. This software includes a program switch that allows the ROV pilot to select which of the two systems is used to control the manipulator, and to switch the control from one to the other during operation. Figure 1 shows a block diagram of the dual manipulator ROV control system architecture which includes the developed collision avoidance algorithm.

The proposed collision-free manipulation algorithm is an on-line method based on a voxel map—a representation of Cartesian space discretised into a regular grid. The work of [11] inspires the core idea of the algorithm. It is a purely kinematical method that processes kinematic parameters (joint positions) provided by a command control system and returns collision-free kinematic parameters in the same form, which are forwarded to the existing low-level joint space motion controller. Kinematic modelling was essential for the algorithm development, and it had previously been derived by Sivčev et al. [33].

The Cartesian space occupied by manipulators and obstacles is discretised into a regular grid of cubic voxels with the desired spatial resolution, determined by the voxel size. The smaller the voxels are, the more accurate the modelling will be. The maximum total number of voxels *n* in the grid is inversely proportional to the voxel size *s* and grows according to the cubic law in (1).

(1) $$n=\frac{1}{s^{3}}\;\;\;\mathrm{for}\;\;\;s=\left(0,\ldots ,1\right]$$

A larger voxel grid leads to a higher computational load for the collision avoidance algorithm. This used to be a relevant factor in the past, but with the processing power currently available it is not anymore, as will be seen from the simulation and experimental results.

Voxel maps are formed by highlighting those voxels that are occupied by worksite objects. Maps that represent static obstacles only need to be computed once, during the initial stage of the algorithm. On the other hand, voxel maps that represent manipulators and other dynamic obstacles have to be recomputed in the control loop iteration. It is assumed that the geometry of worksite objects is known, i.e., that the CAD models of obstacles and manipulators are available. Each static obstacle’s pose, as well as manipulator poses, are also assumed to be known. The common reference frame in this case is a fixed coordinate frame on the base vehicle (ROV). Collision detection is done by checking whether more than one object occupies the same voxel at a given point in time.

### 2.1. Voxel Map Modelling—Static Obstacles

For each object, a separate voxel map can be generated based on its CAD model. An alternative way is to construct multiple object CAD assemblies and create a voxel map for each one or form a single assembly that comprises all static obstacles and transform it into a voxel map. For each mission an ROV can be equipped with a slightly different set of devices; additionally, the same devices can be mounted in different locations. Having an independent voxel map for each device, or groups of devices that are often used together, it is straightforward to create a single final voxel map for each mission as a union of separate voxel maps. Therefore, for the sake of modularity, it is preferable to address each object separately when constructing voxel maps.

**Figure 2:**
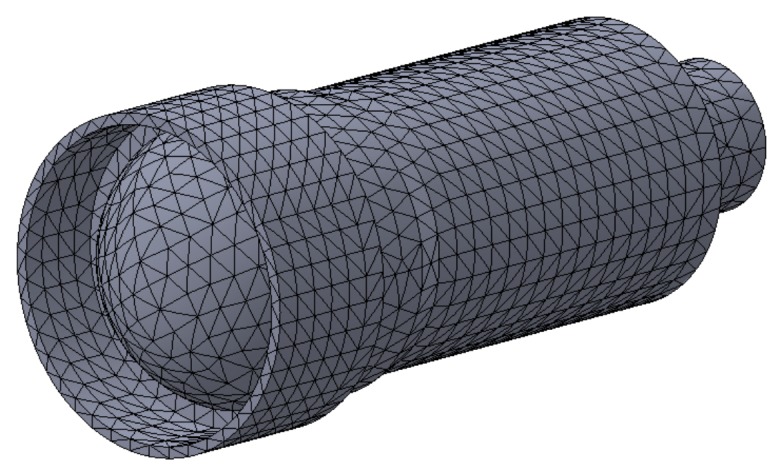
Meshing an ROV camera CAD model into a point cloud using SolidWorks.

The first step is transforming an obstacle’s CAD model into a point cloud, which can be achieved using most 3D CAD tools. Using SolidWorks for example, this can be done by generating a mesh of points with a user-specified mesh density for each surface of the CAD model (Figure 2). Selecting an appropriate mesh density is crucial for avoiding gaps in voxel maps—the Euclidean distance between any two points in the mesh should be at least an order of magnitude smaller than the voxel size.

This method does not consider the object’s volume, but only its external surface. Therefore, the resulting point cloud is a shell of the shape of the object. The method used to compensate for the loss of information about the interior of the obstacle is performed on the voxel map level and will be described later in this section. The resulting point cloud is a set of *m* data points $P_{i}$ defined with Cartesian coordinates:

(2) $$P=\{P_{i}\left(x_{i},y_{i},z_{i}\right)\in R^{3}\;|\;i=1,\ldots ,m\}$$

Choosing the point cloud reference frame is essential—it has to be either the ROV base frame $O_{xyz}$ or any frame ${}^{x}O_{xyz}$ whose pose $H_{x}$ relative to the base is known or possible to measure. In the latter case, expressing the point cloud in the ROV base frame is straightforward:

(3) $$P=H_{x}{}^{x}P$$

**Figure 3:**
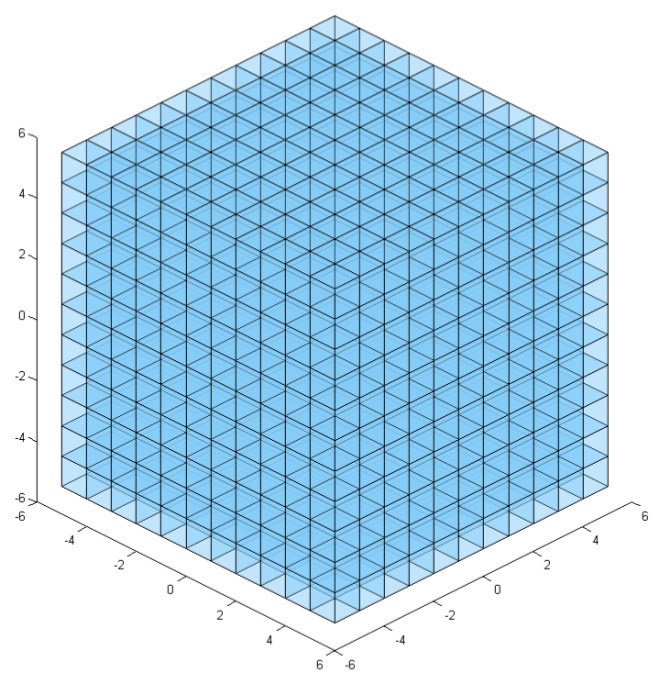
Regular 3D voxel grid—unoccupied.

The next step is mapping the generated point cloud to voxels in the Cartesian grid. Voxels occupied by at least one point are highlighted and included into the voxel map. This is done by finding the closest voxel to each point, i.e., the voxel with the smallest Euclidean distance from the point. A regular voxel grid V (Figure 3) is given by:

(4) $$V=\{V_{i}\left(x_{i},y_{i},z_{i}\right)\in R^{3}|\;i=1,\ldots ,n\}$$

where each voxel is defined with Cartesian coordinates representing its volumetric centroid.

Mapping each point in the point cloud P to its nearest voxel is performed by a rounding function, given by:

(5) $${\hat{P}}_{i}=\lfloor \frac{\left(P_{i}+\frac{s}{2}\right)}{s}\rfloor s\;\;\;\mathrm{for}\;\;\;i=1,\ldots ,m$$

As a result, each point in the newly formed point cloud $\hat{P}$ gets pushed to its nearest voxel. The coordinates of these points are used to highlight voxels in the grid and form a voxel map that represent the object’s shell. The next step is to compensate for the lost information on the object’s interior by filling the cavities. This is done by identifying all internal voxels and marking them as occupied, see Algorithm 1. The result is a voxel map that models the entire volume of the object. The same process is repeated for all other obstacles.

Finally, a single voxel map representing all static worksite objects is created as a union of separate obstacle voxel maps. Since this voxel map is constant, it has to be constructed only once before the execution of the collision avoidance algorithm.
**Algorithm 1** Algorithm for supplementing an object shell voxel map with internal volume voxels1:**procedure** FillInternalVolume(V)2:    **for all** z axis **do**3:        cnt←04:        **for all** x axis **do**5:           **for all** y axis **do**6:               **if** V(x,y,z)=1 **then**7:                   cnt←cnt+18:                   **if** cnt=1 **then**9:                       $V_{1}\leftarrow V\left(x,y,z\right)$10:                       $y_{1}\leftarrow y$11:                   **else if** cnt=2 **then**12:                       **if** $y-y_{1}=V_{size}$ **then**13:                          $V_{1}\leftarrow V\left(x,y,z\right)$14:                          $y_{1}\leftarrow y$15:                          cnt←cnt-116:                       **else**17:                          $V_{2}\leftarrow V_{1}$18:                          $V_{1}\leftarrow V\left(x,y,z\right)$19:                          cnt←cnt+120:                          **for** $y\leftarrow y_{1},y_{2}$ **do**21:                              V(x,y,z)←122:                          **end for**23:                       **end if**24:                   **end if**25:               **else if** cnt≥2 **then**26:                   cnt←027:               **end if**28:           **end for**29:        **end for**30:        cnt←031:        **for all** y axis **do**32:           **for all** x axis **do**33:               **if** V(x,y,z)=1 **then**34:                   cnt←cnt+135:                   **if** cnt=1 **then**36:                       $V_{1}\leftarrow V\left(x,y,z\right)$37:                       $y_{1}\leftarrow y$38:                   **else if** cnt=2 **then**39:                       **if** $x-x_{1}=V_{size}$ **then**40:                          $V_{1}\leftarrow V\left(x,y,z\right)$41:                          $x_{1}\leftarrow y$42:                          cnt←cnt-143:                       **else**44:                          $V_{2}\leftarrow V_{1}$45:                          $V_{1}\leftarrow V\left(x,y,z\right)$46:                          cnt←cnt+147:                          **for** $x\leftarrow x_{1},x_{2}$ **do**48:                              V(x,y,z)←149:                          **end for**50:                       **end if**51:                   **end if**52:               **else if** cnt≥2 **then**53:                   cnt←054:               **end if**55:           **end for**56:        **end for**57:    **end for**58:    **return** V59:
**end procedure**



### 2.2. Voxel Map Modelling—Manipulators

The method for creating a voxel map representing a manipulator is slightly different due to its moving parts. The manipulator consists of a base and its links. Since the base is fixed, it is modelled as a static object and incorporated in the static obstacle voxel map. The situation with links is different as they move in space. Determining the volume in space occupied by each link requires a kinematic model of the manipulator, a CAD model of each of the links, and the manipulator’s latest angular joint positions acquired in each control loop. This volume can be modelled as a point cloud and transformed into a voxel map. A more efficient way is to precompute voxel maps for each manipulator links from their CAD models, and then iteratively, using the kinematic model and angular joint positions, remap the voxel maps appropriately. The kinematic model is derived according to the Denavit-Hartenberg (DH) convention for attaching reference frames to the links of a manipulator [33]. The addressed manipulator consists of six joints numbered from 1 to 6, and seven links numbered from 0 to 6, starting from the base. Each link has a coordinate frame rigidly attached to it; its location is determined by the DH convention.

**Figure 4:**
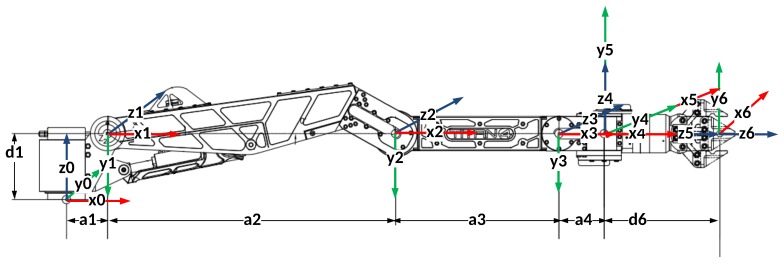
Kinematic model of a Schilling Titan 2 manipulator.

The first step in creating a manipulator voxel map is to create a voxel map for each link by using the technique for a separate static object described in Section 2.1. However, in the step of transforming a CAD model into a point cloud, it is essential to choose the appropriate reference coordinate frame for each link. These frames have to be the coordinate frames rigidly attached to the links according to the DH convention (Figure 4).

Thus, the resulting voxel map ${}^{k}L$ for the $k^{\mathrm{th}}$ link (k=1,..,6) expressed in the coordinate frame ${}^{k}O_{xyz}$, is given by:

(6) $${}^{k}L={\{}^{k}L_{i}\left(x_{i},y_{i},z_{i}\right)\in R^{3}|i=1,..{,}^{k}n\}$$

where ${}^{k}n$ is the number of voxels that describe the $k^{\mathrm{th}}$ link. The pose of the coordinate frame ${}^{k}O_{xyz}$ can be expressed in the manipulator base frame ${}^{0}O_{xyz}$, as a homogeneous transformation calculated with the standard forward kinematics equation using the appropriate joint position values q, given by [34]:

(7) $$H_{k}^{0}=\prod_{i=1}^{k}T_{i}^{i-1}\left(q_{i}\right)$$

This resulting homogeneous transformation can be expressed in the ROV base frame:

(8) $$H_{k}=H_{0}H_{k}^{0}$$

where $H_{0}$ is the pose of the manipulator base in the ROV frame. Finally, a voxel map for the *k*th link expressed in the ROV base coordinate frame $O_{xyz}$ is acquired by multiplying each voxel from the voxel map given in (6) with the homogeneous transformation given in (8):

(9) $${{}^{0}L_{k}}_{i}=H_{k}{{}^{k}L_{k}}_{i}\;\;\;\mathrm{for}\;\;\;i=1,..{,}^{k}n$$

The same procedure is repeated for each of the manipulator’s links, excluding the base. Subsequently, a single manipulator voxel map is created as the union of the individual links’ voxel maps. The voxel map derived in this way determines which voxels the manipulator occupies for any given joint configuration.

Obstacles independent of the ROV base vehicle are not addressed in this paper. Examples of such obstacles related to ROV operations are the sea floor, a dock wall, or any offshore infrastructure an ROV is not supposed to collide with while operating in its vicinity. Regardless of whether they are stationary or moving, these can be considered dynamic since the ROV is in motion; unless ideal station keeping is assumed. Computer vision might be a potential solution to identifying these obstacles and generating corresponding point clouds. Having the point cloud, transferring it into a voxel map is a straightforward process. Alternatively, if the obstacle structure is known, a method similar to that used for manipulator links can be applied. In this case, the missing component, to be potentially implemented leveraging computer vision, would be the estimation of the obstacle’s pose relative to the ROV. The up-to-date software solution we present is applicable only for detecting collisions between the moving manipulators and the static workspace obstacles.

### 2.3. Voxel Map Modelling—Manipulators’ Workspaces

The static obstacle voxel map described earlier in Section 2.1 is formed based on the CAD models of all objects with which manipulators are not supposed to collide, without considering the manipulators’ workspace size, and therefore regardless of whether the manipulators can reach the potential obstacles. If static obstacles are out of the manipulators’ reachable workspace, there is no reason to include them in the voxel map. However, as the two manipulators’ workspaces do not overlap entirely, some obstacles are reachable by both manipulators and some only by one of them. Instead of using a single voxel map comprising of all obstacles, two separate voxel maps are used, one for each manipulator, each of which contains only the objects that are reachable by that manipulator. This section describes the procedure to generate these two voxel maps.

The Cartesian space defining a manipulator’s reachable workspace has to be transformed into a voxel map. The first step to create a manipulator workspace voxel map is discretising the manipulator’s configuration space (C-space) and transforming it into the Cartesian space. The C-space represents the set of all allowable transformations of the manipulator; for a 6 degrees-of-freedom (DOF) manipulator it forms a 6-dimensional manifold [35]. For each DOF of the manipulator (k=1,…,6), a number of intervals along the generalised joint coordinate $q_{k}$ is specified as:

(10) $$N_{k}=\lfloor \frac{q_{k}^{max}-q_{k}^{min}}{\Delta q_{k}}\rfloor$$

where $q_{k}^{min}$ and $q_{k}^{max}$ are the physical limits of the $k^{\mathrm{th}}$ joint motion, and $\Delta q_{k}$ is the discretisation resolution of the $k^{\mathrm{th}}$ joint. The most straightforward discretisation method is uniform discretisation—fixing $\Delta q_{k}$ to a constant value used for all joints. The deficiency of this approach is that the Cartesian points it generates are not equidistant. This can be improved using advanced discretisation methods such as heuristic and optimal discretisation [13]. However, creating a workspace voxel map does not require having a dense point cloud of equidistant reachable end-effector Cartesian points throughout the entire workspace volume. Nevertheless, accurate and sufficiently dense modelling of the outer shell of the working space is required. As the C-space discretisation takes place only once, the computational time for this step is not of importance, and therefore the uniform discretisation method with high enough resolution is sufficient. Discretisation of the C-space generates *K* joint configuration vectors q, where $K=\prod_{k=1}^{6}N_{k}$, which are transformed into the Cartesian space using standard forward kinematics equation [34], resulting in a point cloud expressed in the manipulator’s base frame:

(11) $${}^{0}W=\left\{{}^{0}W_{j}=\prod_{i=1}^{6}T_{i}^{i-1}\left(q_{i}\right)\;|\;j=1,\ldots ,K\right\}$$

Using the same process that was used for generating the static obstacles map, this point cloud is transformed into a voxel map and expressed in the ROV base frame W, see Equations (2)–(5). Since the resulting manipulator workspace voxel map might contain gaps within the volume, the missing voxels are added using Algorithm 1. Finally, the resulting voxel map represents the entire volume of the manipulator’s workspace. The intersection between this voxel map and the static obstacle voxel map then results in a map $O_{1}$ which is comprised only of obstacles reachable by that manipulator. The same procedure is repeated for the other manipulator, yielding voxel map $O_{2}$. These newly formed static obstacle voxel maps are smaller in size which is convenient for storage and computation.

### 2.4. The Collision Avoidance Algorithm

Regardless of the manipulator control input device, mode of operation (manual, semi-automatic or fully automatic), and operational space (joint or Cartesian), the output kinematic parameters to be supplied to the low-level manipulator motion controller are assumed to be angular joint positions. Additionally, it is considered that the desired joint position is a continuous digital signal. The algorithm requires having access to desired motion commands and current joint position sensor measurements for both manipulators in each control loop. After the manipulator motion commands are issued, the desired joint position vectors are processed by the collision avoidance algorithm based on the procedure described in this section.

The first step in implementing the algorithm is forming a path between initial and desired joint configurations. Since the difference between the corresponding values of desired and initial joint positions is assumed to be relatively small, the number of steps forming this path does not have to be large. This provides a sequence of specific manipulator poses in space-time, for each of which a manipulator voxel map is created, using the technique described in Section 2.1, Section 2.2, and Section 2.3. The resulting maps are merged into a single map, as a union between them. The newly formed voxel map ($M_{D1}$) represents the entire volume that the manipulator would sweep moving from the initial to the desired pose. The same process is repeated for the other manipulator and the voxel map $M_{D2}$ is formed. The next step is constructing voxel maps that represent the currently occupied workspace. To do this, the manipulator voxel map for the second manipulator $M_{C2}$ is created based on its current pose and merged with the static obstacle voxel map for the first manipulator ($O_{1}$) as a union between them, forming a new map $O_{M1}$. Using the same procedure, map $O_{M2}$ is generated for the other manipulator. The final step is constructing two collision voxel maps, one for each manipulator. The first one is formed as an intersection between $M_{D1}$ and $O_{M1}$. If the resulting collision voxel map is empty, the motion is regarded as collision-free and the desired motion command is forwarded to the low-level motion controller of the manipulator. However, if there is a single voxel occupied in the resulting collision voxel map, the impending collision is detected. In that case, the desired joint position vector supplied as a motion command is ignored and the current joint position vector, which represents the collision-free configuration, is forwarded to the low-level motion controller of the manipulator. This provides an answer whether the first manipulator by moving to the desired pose is going cause a collision with the second manipulator or static obstacles. The second collision map is formed in the same way using $M_{D2}$ and $O_{M2}$. This algorithm is repeated throughout the entire duration of the manipulators’ operation.

## 3. Simulation and Experimental Results

**Figure 5:**
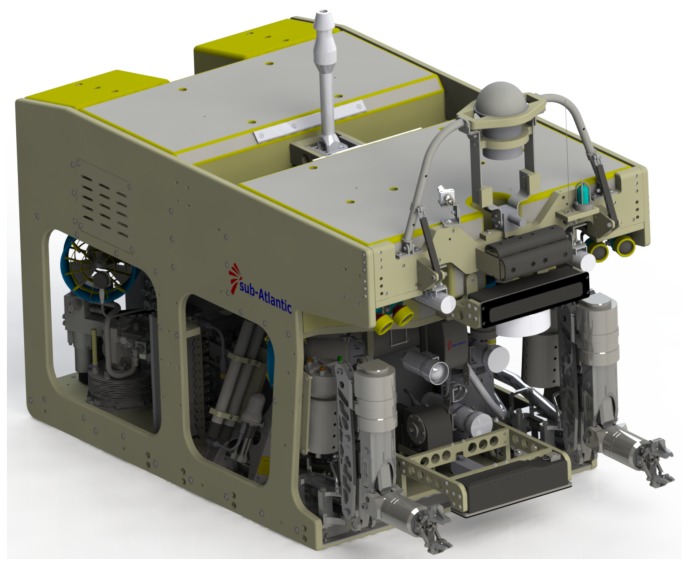
University of Limerick MRE ROV.

In this section, the authors analyse an implementation scenario for the proposed collision-free manipulation algorithm on an ROV equipped with two manipulators. One such robotic vehicle is the MRE ROV (Figure 5), a University of Limerick owned ROV which is mainly used for research purposes.

**Figure 6:**
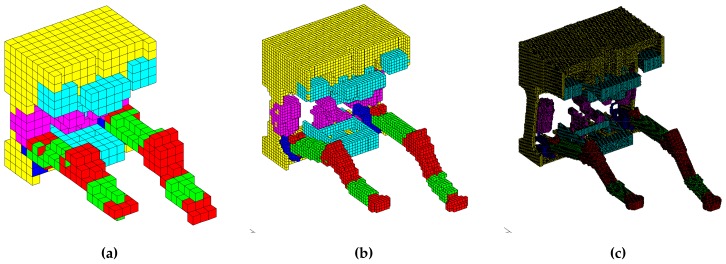
MRE ROV modelled with voxels of different size: (**a**) 100 mm; (**b**) 33 mm; and (**c**) 10 mm.

This ROV is equipped with two seven function Schilling ORION manipulators, both of which have position sensors in each joint. The information provided by these sensors along with the known CAD model of the whole ROV, including the relative pose between the robotic manipulator bases, are sufficient to implement the proposed collision detection algorithm. The collision detection algorithm is developed in C++ in the form of an independent Dynamic-Link Library (DLL) which is encapsulated in the robotic manipulation control software, developed previously using the Matlab Robotics Toolbox and LabView [22]. The simulation scenario for the validation of the developed software, including the proposed collision avoidance algorithm, consists of a mathematical model of the real MRE ROV and the two accompanying manipulators. Two cases are addressed in simulations: the collision between each manipulator and the base vehicle, and the collision between the two manipulators. Additionally, the developed collision-free manipulation algorithm has been tested in a real-world experimental setup in dry laboratory conditions, where a single Schilling Titan 2 manipulator was kept from colliding with the floor using the discussed method. Voxels of different sizes ranging from 10 mm to 100 mm were used for the modelling of all the rigid objects that are part of the ROV, including the manipulators (Figure 6). Such variation enabled analysing how the algorithm behaves with the increase in the computational load due to the voxel map size, that is, how the voxel size affects the algorithm execution time.

**Figure 7:**
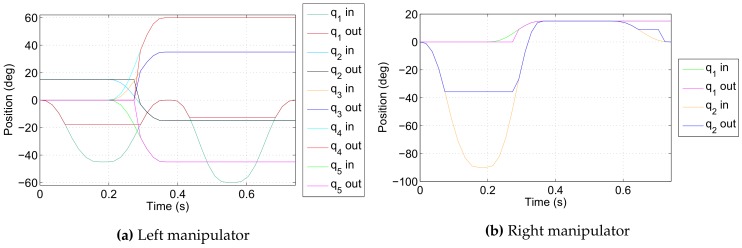
Reference input and collision-free output trajectories in joint space.

**Figure 8:**
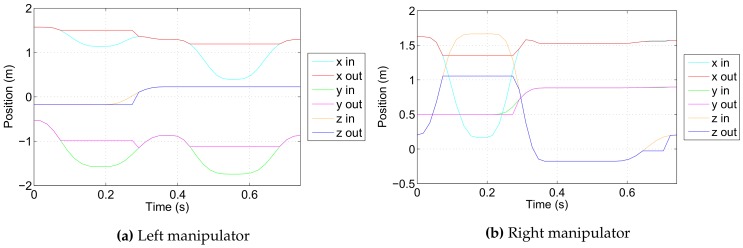
Reference input and collision-free output trajectories in Cartesian space.

The simulation scenario runs as follows. Both robotic manipulators start from predefined collision-free initial configurations defined in joint space; joint space trajectories are then generated for both manipulators simulating the motion command issued by a human operator or a computer program. Each trajectory is a predefined sequence of joint space configurations such that it first causes a collision between the manipulator and the body of the ROV base vehicle and afterwards a collision between the two manipulators. These reference trajectories are inputs of the developed algorithm which acts as a collision filter, sensing and prohibiting any motion that causes a collision and allowing only collision-free motion. The reference input trajectories and resulting output trajectories are given in Figure 7 in joint space and in Figure 8 in Cartesian space; voxel size is 33 mm.

**Figure 9:**
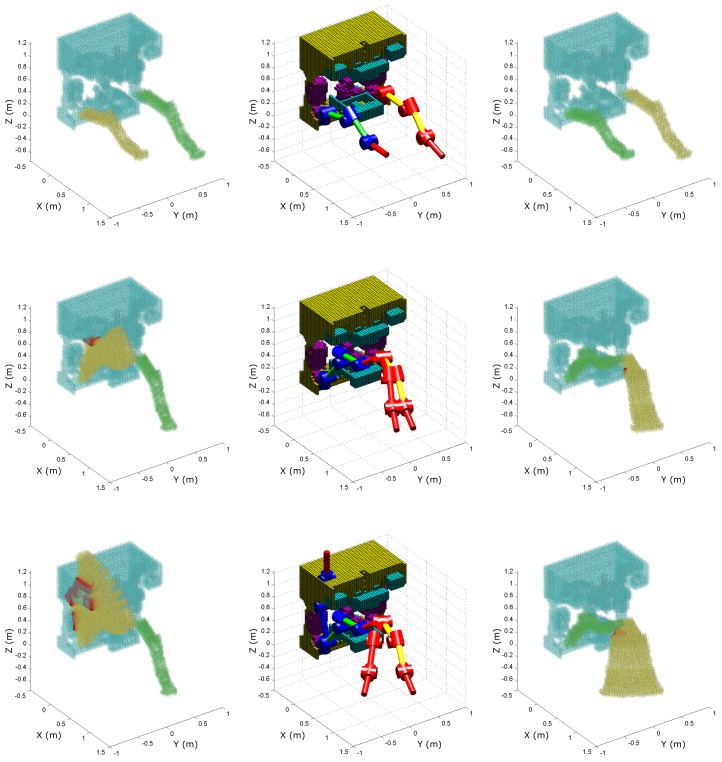
Simulation of the collision detection between each manipulator and the ROV.

**Figure 10:**
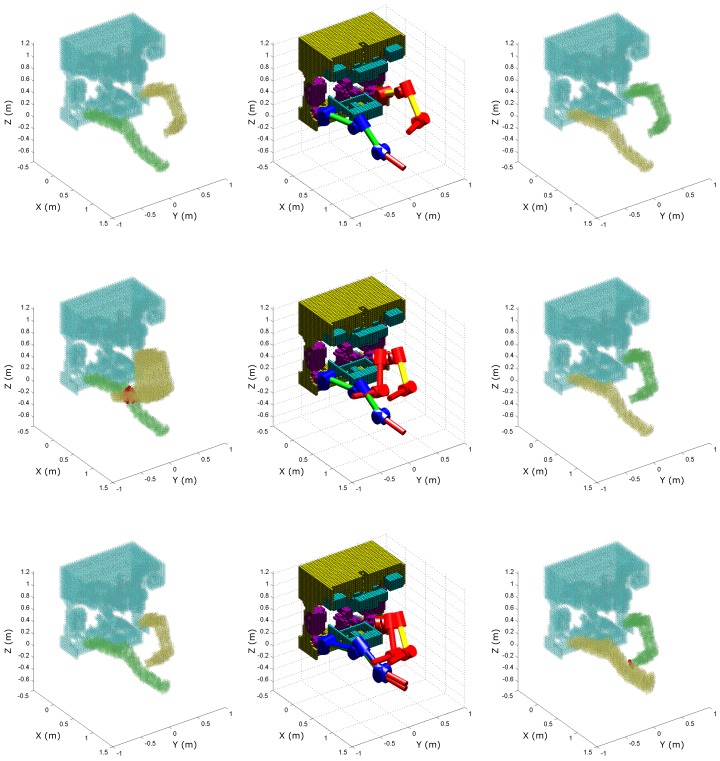
Simulation of the collision detection between two manipulators.

Figure 9 and Figure 10 illustrate the visualisation of the collision detection algorithm for the described simulation scenario.

The red-yellow and blue-green manipulators on the middle images represent the collision-free output motion of the proposed algorithm, while the red and blue manipulators represent the discarded reference motion. In each control loop, the collision detection algorithm checks not only whether the single desired configuration of the reference trajectory leads to collision, but also the configurations in between. That is, the whole volume the manipulator would sweep if it was to move from the current to the desired configuration, which is illustrated by the yellow voxels in the figures. Each control loop executes the collision detection algorithm twice to check the reference motion for each manipulator. Therefore, the green voxels represent the other, passive manipulator, which together with the ROV body (represented by light blue voxels) forms the obstacle voxel map for that iteration. Finally, red voxels represent the imminent collision sensed by the developed algorithm. Table 1 shows the computational load of the collision detection algorithm for different voxel sizes, numbers of voxels, and numbers of intersection operations; as well the time required for different algorithm phases.

**Figure 11:**
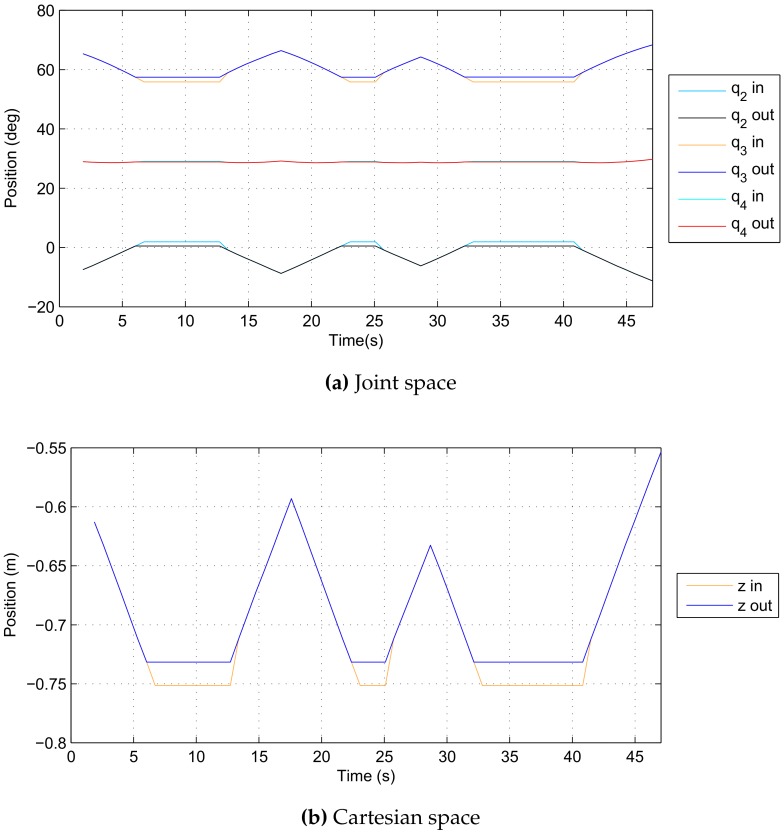
Reference input and collision-free output trajectories from the experiment.

**Figure 12:**
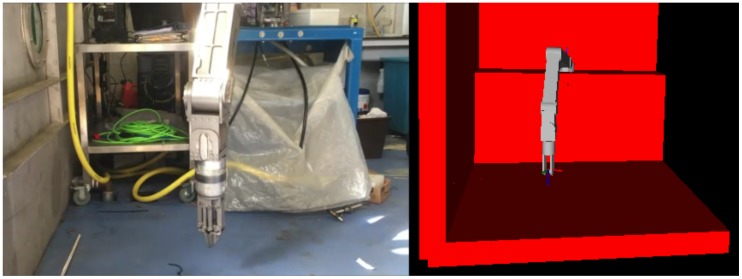
Experimental setup with the floor as an obstacle.

The developed collision detection algorithm has been tested in a real-world experimental setup using a Schilling Titan 2 manipulator. In the addressed scenario, the manipulator was intentionally commanded to collide with the floor, which the collision detection algorithm successfully prohibited. The trajectories utilised in this experiment are depicted in Figure 11, while Figure 12 shows a photo of the experiment.

## 4. Conclusion and Future Work

The proposed collision-free motion algorithm for marine robotic manipulation has been described and successfully evaluated in simulations and experimental setup using a real underwater manipulator. The developed solution can be easily integrated as a software upgrade into the control systems that are present in the global fleet of industry standard work-class ROVs. Tests with real ROV hardware verified that the computational load and memory consumption are not a problem. The authors believe that the presented collision detection algorithm has a potential to be a useful add-on for ROV pilots enabling them to execute typical IRM tasks with greater ease and speed, e.g., handling tools with both manipulators in close vicinity to fragile equipment, such as cameras and sonars, knowing that no harm can be done. This would reduce their fatigue and eventually provide cost savings in subsea IRM operations in oil and gas, the MRE sector, and other fields of application.

Ongoing work is integrating the developed algorithm into the MRE ROV control software and testing it in offshore trials. Subsea experiments will include physical simulation of intervention operations with various mock-up test panels and tool skids. Specific manipulation tasks are to be repeated multiple times with and without the implementation of the collision detection algorithm, where an ROV manipulator operator will focus on executing tasks with increased speed. Measuring the time required to complete the task and number of collisions during the process, and comparing them to the traditional method, will reveal the actual performance of the proposed collision detection. Additionally, further algorithm development is planned in order to address aspects such as software optimisation, detecting potential manipulator self-collision, expanding the model library by including different ROV operated tools, developing a real-time GPU-based visualisation as a pilot assisting tool which can be useful in turbid and low visibility environments, and investigating alternative methods of acquiring an ROV’s point cloud, including camera imaging and laser scanning.

## Figures and Tables

**Table 1:** Computational load analysis of the collision detection algorithm.

Voxel Size (mm)	Manip. Voxels	Obstacle Voxels	Intersection Operations	Manip. Voxeling (ms)	Intersecion (ms)	Total Loop (ms)
100	1120	710	795,200	3.4	0.6	8
66	2500	1688	4,220,000	3.6	1.5	10.2
33	9850	5016	49,407,600	3.8	5.6	18.8
22	18,530	7399	137,103,470	4.5	10.7	30.4
15	47,610	9964	474,386,040	5.8	26.9	65.4
10	102,030	13,530	1,380,465,900	8	58.8	133.6

## Supplementary Materials

The following videos are available online: Figure 9 and Figure 10: Simulation of collision-free manipulation algorithm for work-class ROVs at https://vimeo.com/259666778, and Figure 12: Collision-free manipulation algorithm experiment at https://vimeo.com/259666780.

## Abbreviations

The following abbreviations are used in this manuscript:

ROV: Remotely Operated Vehicle
IRM: Inspection, Repair, and Maintenance
MRE: Marine Renewable Energy
MCU: Master Control Unit
DOF: Degree of Freedom
DLL: Dynamic-link Library
GPU: Graphics Processing Unit
CAD: Computer-aided Design
